# LONELINESS, SOCIAL ISOLATION, AND DOMAINS OF COGNITIVE IMPAIRMENT IN THE ENGLISH LONGITUDINAL STUDY OF AGEING

**DOI:** 10.1093/geroni/igz038.684

**Published:** 2019-11-08

**Authors:** Jessica G Abell, Jessica Abell, Dorina Cadar, David J Llewellyn, Andrew Steptoe

**Affiliations:** 1 University College London, London, United Kingdom; 2 University College London, London, England, United Kingdom; 3 University of Exeter Medical School, Exeter, England, United Kingdom

## Abstract

Globally the numbers of older people who live alone and those who may experience certain risk factors have risen. In this study, we aim to examine associations between social isolation and loneliness with different domains of cognitive impairment. Data are from the English Longitudinal Study of Ageing (ELSA). Social isolation and loneliness were measured in 2012-2013 and cognition in 2017-2018, using the Harmonised Cognitive Assessment Protocol (HCAP) in 1,200 men and women aged ≥65 years. General cognitive impairment was measured using the Mini-Mental Status Examination (MMSE); memory was assessed using the CERAD word list, attention & speed using the Symbol-Digit Modalities Test and executive function by a number series test. Loneliness, measured using the UCLA scale, was associated with a higher risk of neurocognitive impairment (MMSE<24), lower memory scores, poorer attention and executive function. However, social isolation was only found only to be associated with lower levels of memory.

